# Sickle Erythrocytes Target Cytotoxics to Hypoxic Tumor Microvessels and Potentiate a Tumoricidal Response

**DOI:** 10.1371/journal.pone.0052543

**Published:** 2013-01-09

**Authors:** David S. Terman, Benjamin L. Viglianti, Rahima Zennadi, Diane Fels, Richard J. Boruta, Hong Yuan, Mathew R. Dreher, Gerald Grant, Zahid N. Rabbani, Ejung Moon, Lan Lan, Joseph Eble, Yiting Cao, Brian Sorg, Kathleen Ashcraft, Greg Palmer, Marilyn J. Telen, Mark W. Dewhirst

**Affiliations:** 1 Molecular Genetics Program, Jenomic, Carmel, California, United States of America; 2 Department of Radiology, University of Michigan, Ann Arbor, Michigan, United States of America; 3 Department of Medicine, Division of Hematology, Duke University Medical Center, Durham, North Carolina, United States of America; 4 Department of Radiation Oncology, Duke University Medical Center, Durham, North Carolina, United States of America; 5 Department of Pediatrics, University of North Carolina, Chapel Hill, North Carolina, United States of America; 6 Department of Radiology, University of North Carolina, Chapel Hill, North Carolina, United States of America; 7 National Institutes of Health, Clinical Center, Diagnostic Radiology Department, Bethesda, Maryland, United States of America; 8 Department of Surgery, Division of Neurosurgery, Duke University Medical Center, Durham, North Carolina, United States of America; 9 Department of Biostatistics, Duke University Medical Center, Durham, North Carolina, United States of America; 10 Department of Radiology, Mayo Clinic Foundation, Rochester, Minnesota, United States of America; 11 Department of Surgery, Division of Neurooncology, Duke University Medical Center, Durham, North Carolina, United States of America; 12 Cancer Diagnosis Program, National Cancer Institute, Bethesda, Maryland, United States of America; University of Chicago, United States of America

## Abstract

Resistance of hypoxic solid tumor niches to chemotherapy and radiotherapy remains a major scientific challenge that calls for conceptually new approaches. Here we exploit a hitherto unrecognized ability of sickled erythrocytes (SSRBCs) but not normal RBCs (NLRBCs) to selectively target hypoxic tumor vascular microenviroment and induce diffuse vaso-occlusion. Within minutes after injection SSRBCs, but not NLRBCs, home and adhere to hypoxic 4T1 tumor vasculature with hemoglobin saturation levels at or below 10% that are distributed over 70% of the tumor space. The bound SSRBCs thereupon form microaggregates that obstruct/occlude up to 88% of tumor microvessels. Importantly, SSRBCs, but not normal RBCs, combined with exogenous prooxidant zinc protoporphyrin (ZnPP) induce a potent tumoricidal response via a mutual potentiating mechanism. In a clonogenic tumor cell survival assay, SSRBC surrogate hemin, along with H_2_O_2_ and ZnPP demonstrate a similar mutual potentiation and tumoricidal effect. In contrast to existing treatments directed only to the hypoxic tumor cell, the present approach targets the hypoxic tumor vascular environment and induces injury to both tumor microvessels and tumor cells using intrinsic SSRBC-derived oxidants and locally generated ROS. Thus, the SSRBC appears to be a potent new tool for treatment of hypoxic solid tumors, which are notable for their resistance to existing cancer treatments.

## Introduction

Hypoxic tumor cells are present in the vast majority of human solid cancers and establish significant niches for therapeutic resistance and tumor recurrence. Under hypoxic conditions within tumors, evolutionarily conserved oxygen sensors initiate distress pathways that lead to activation of hypoxia-inducible transcription factors, proinflammatory and pro-angiogenic stimuli [1]–[3]. The latter induce a disordered network of blood vessels, anastomotic branches, fenestrations and shunts resulting in heterogeneous blood perfusion, nutrient delivery, cyclic or chronic deoxygenation and aerobic glycolysis [4]. In this microenvironment, tumors exhibit impaired drug transport, treatment resistance and aggressive malignant progression.

Therapeutic attempts to selectively target hypoxic tumor cells have largely focused on bioreductive prodrugs that are activated by enzymatic reduction under moderate to severe hypoxic conditions. To date, these agents have not proven clinically useful [5]. Tirapazamine, the earliest prototype of this group demonstrated no survival benefit when added to standard chemotherapy and was associated with dose limiting myelosuppression related to activation of aerobic reductases in normal tissues [6]. Although the key vulnerabilities of hypoxic cells are not yet determined, a second approach uses small molecule inhibitors targeting hypoxia-inducible factor 1 (HIF1), the unfolded protein response (UPR) and mTOR pathways [5]. Both bioreductive and molecularly targeted agents share the challenge of drug penetration through poorly perfused hypoxic tissue [7],[8]. The bioreductive agents must further deal with cumulative toxicity of their DNA-reactive cytotoxins when used together with standard chemotherapeutics [5]. Finally because of the heterogeneity in hypoxia between tumours of the same type, both groups require in vivo diagnostics to accurately measure hypoxia in order to select patients who can benefit most from these treatments [9]. In view of these barriers, conceptually new strategies and agents are needed. In one such approach that differs fundamentally from those directed to hypoxic tumor cells, we herein provide sickle erythrocytes (SSRBCs) to target the hypoxic tumor vascular microenviroment and induce a tumoricidal response using intrinsic SSRBC oxidants and locally generated ROS. Importantly, this approach has little effect on normal vasculature and lacks cumulative toxicity with other cytotoxics suggesting that it may possess a broader therapeutic index than agents that selectively target hypoxic tumor cells alone.

In sickle cell disease, a monogenic mutation in the β-chain of hemoglobin wherein the sixth amino acid in the β-globin chain is changed from glutamic acid to valine, induces hemoglobin polymerization and changes in erythrocyte morphology during hemoglobin desaturation [10]. Disturbances resulting from this mutation include impaired microvascular blood flow [11], episodic vasoocclusion [12], ischemia-reperfusion injury [13], and endothelial cell activation [14],[15]. Tissue hypoxia and deoxygenation of SS hemoglobin occur frequently in sickle cell disease, particularly in venules, where blood velocity is reduced [11],[16]. Hypoxia, oxidative stress and proinflammatory cytokines also upregulate several vascular adhesion receptors [17]–[24]. In response to hypoxia, transgenic sickle mice show pronounced vascular inflammation compared with normal mice, leading to reduced blood flow and transient venular stasis [24],[25]. In the course of painful sickle cell crisis, activated SSRBCs adhere to adhesive ligands on the upregulated vascular endothelium, recruit leukocytes and platelets leading to microvessel occlusion. SSRBCs entrapped in this process undergo autohemolysis generating excessive reactive oxygen species and autooxidized heme iron that results in severe tissue injury [26].

In a parallel to painful sickle cell crisis, the microvasculature of most solid tumors is upregulated to express several vascular adhesion molecules in response to cyclic hypoxia within the tumor [2] and proinflammatory cytokines generated by tumor cells [22],[24],[27]–[30].

These findings provided a conceptual basis for a seminal report which precisely identified a central role for SSRBCs in targeting the upregulated/hypoxic tumor vasculature, inducing vaso-occlusion/autohemolysis and generating intrinsic/locally-derived oxidants leading to endothelial injury and a tumoricidal response [31]. Subsequently, SSRBCs were imaged in tumors or identified in tumor microvessels at autopsy but there were no reported therapeutic applications [32]–[34]. Here, we examine the original concept and demonstrate novel properties of SSRBCs in selectively targeting the hypoxic vascular microenvironment of solid tumors, inducing diffuse tumor vascular occlusion and potentiating the tumoricidal effectiveness of exogenous pro-oxidants both *in vivo* and in vitro.

## Results

### 4T1 mammary carcinoma is neovascularized, hypoxic and expresses several adhesion molecules and heme oxygenase

**Figure 1:**
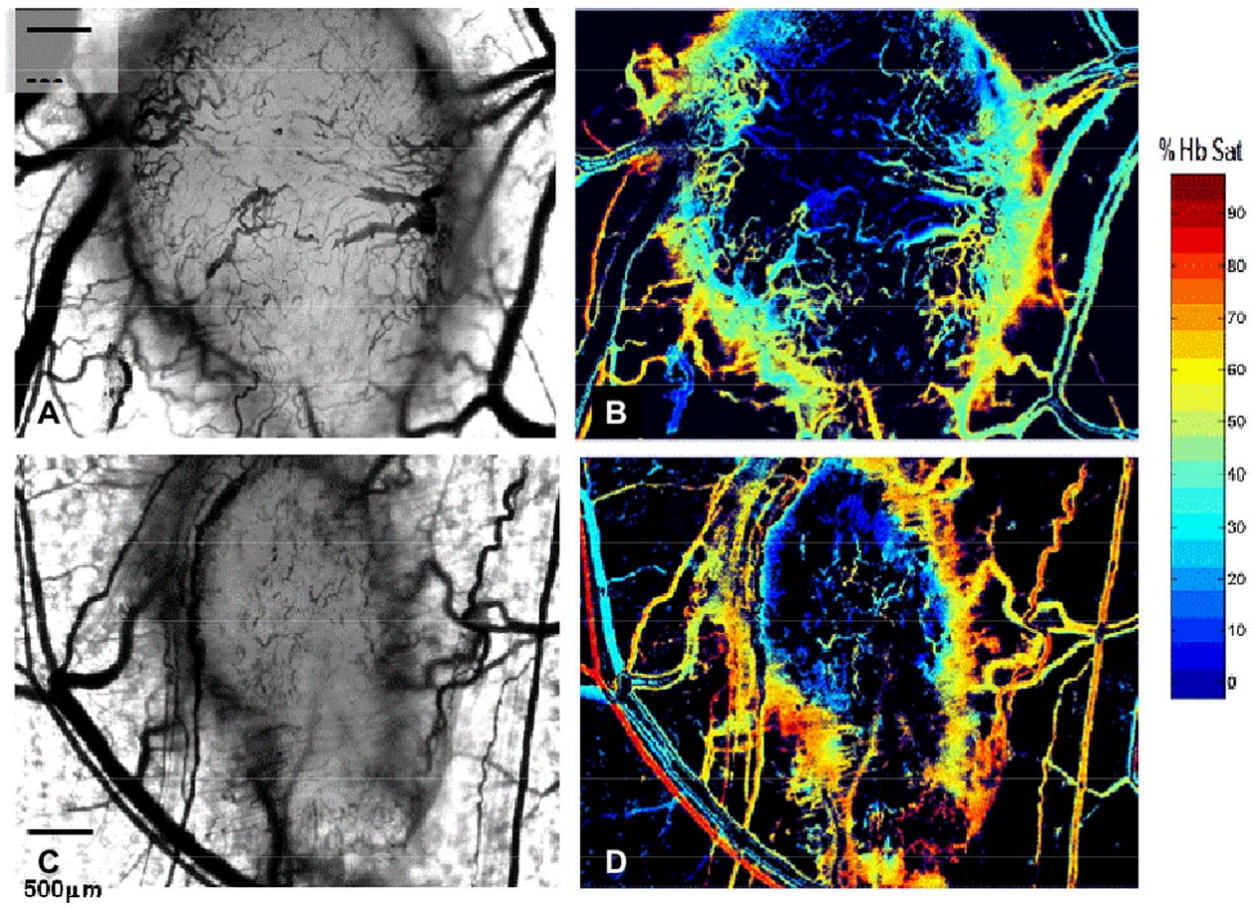
Eight day old 4T1 carcinoma is vascularized and hypoxic. Intravital microscopy of two eight day old 4T1 tumors implanted in the dorsal skin window chamber viewed with light microscopy shows diffuse tumor microvascularity (panels A, C). Corresponding hyperspectral imaging of the same tumors exhibits hemoglobin saturations ≤10% over a 70% of the tumor surfaces (B,D). Magnification 5×.

**Figure 2:**
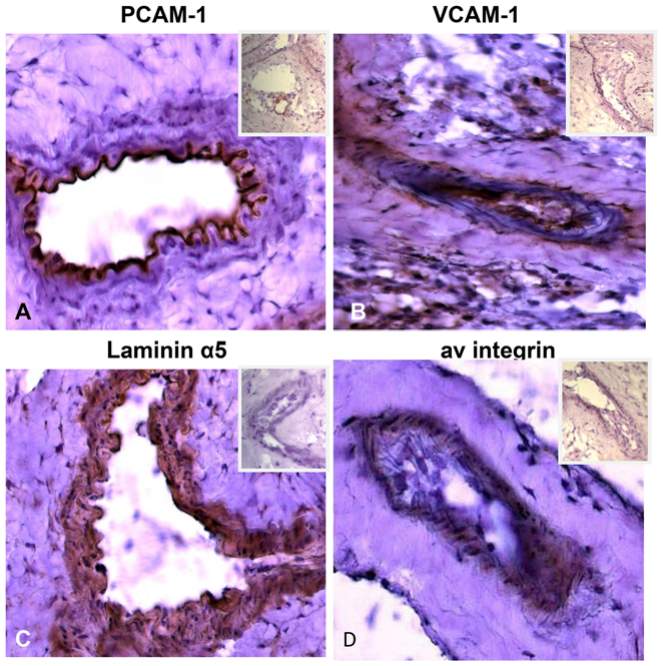
Expression of adhesion molecules on 4T1 tumor vascular endothelium. Frozen sections of 4T1 tumors stained with antibodies against various adhesion molecules shows significant endothelial expression of PECAM-1 (A), ICAM-4 (B), laminin α5 (C). αv integrin (D). Secondary antibodies alone used as negative controls to stain the same tumor sections are shown in the inset of each panel. Magnification 40×.

Initially, we studied the 4T1 carcinoma implanted in the dorsal skin fold window chambers 8 days after tumor implantation for evidence of neovascularization hypoxia, adhesion molecule and heme oxygenase expression. In Figure 1 A,C, eight-day old 4T1 tumors exhibit a dense, disordered vascular network with acutely branching capillaries and anastomotic channels. At this point, the 4T1 tumor vascular microenvironment is markedly hypoxic, evidenced by hemoglobin saturation levels at or below 10% that are distributed over 70% of the tumor space (Figure 1B,D). In addition, tumor microvessels within 4T1 tumors exhibit expression of adhesion ligands PCAM-1, VCAM-1, laminin α5 and av integrins (Figure 2A–D). We also note increased expression of heme oxygenase (HO-1) in 4T1 tumors compared to syngeneic liver cells (Figure S1). Heme oxygenase protects cells against the cytotoxic effect of heme and related oxidation products and is relevant because heme is known to be released by hemolysis during SSRBC-induced vaso-occlusion as described below. Based on these studies, intravital microscopy studies using SSRBCs and NLRBCs described below were conducted on 8-day old 4T1 tumors which are neovascularized, hypoxic and express several adhesion molecules along with heme oxygenase.

### SS RBCs but not normal RBCs accumulate preferentially in tumors and occlude tumor microvessels in vivo

**Figure 3:**
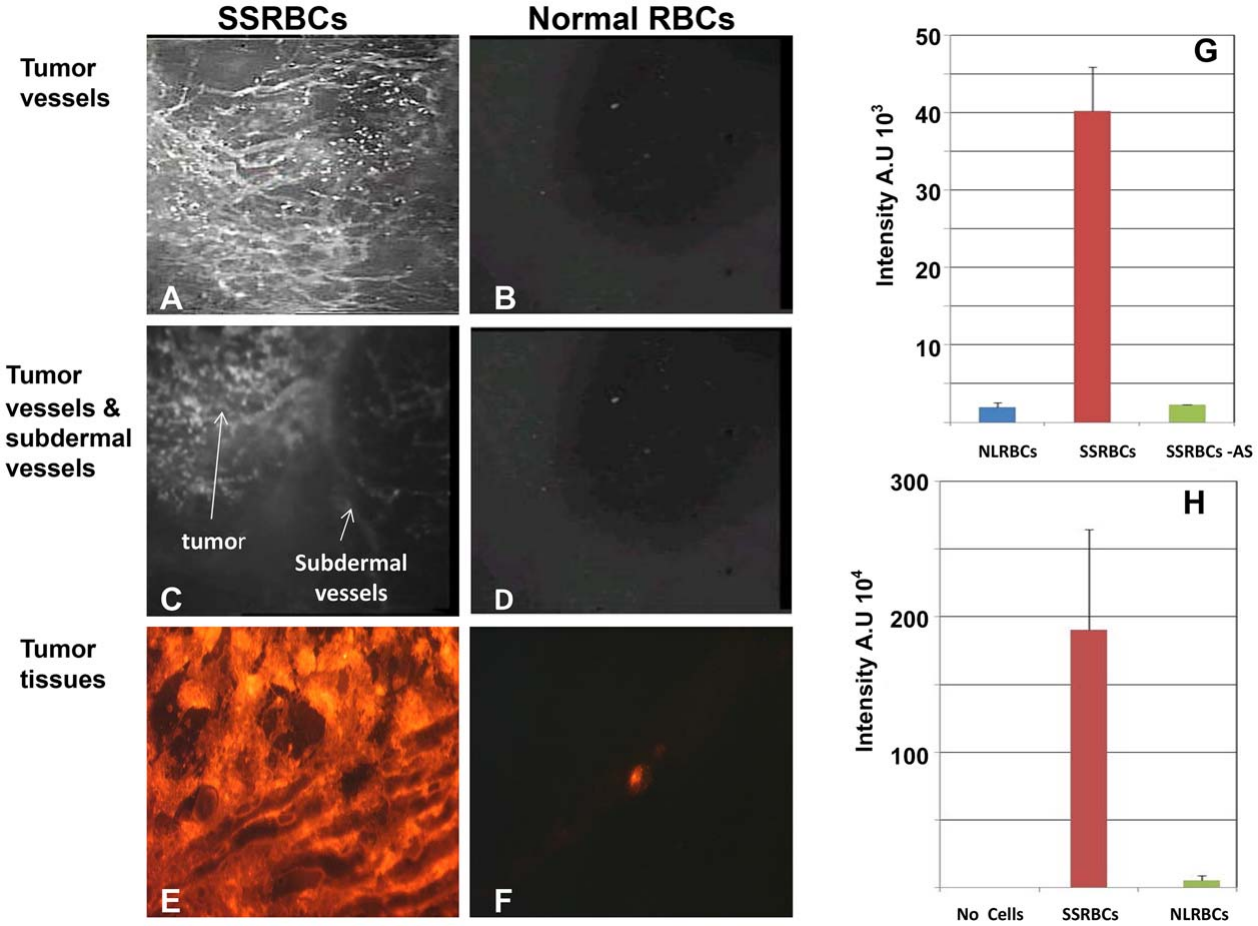
SSRBCs but not NLRBCs accumulate in tumor microvessels within 30 minutes after injection. Intravital microscopy of the vasculature of 8-day old 4T1 tumors implanted in the dorsal skin window chamber within 30 minutes after infusion of mice with SSRBCs (A, C, E) or NLRBCs (B,D,F) shows the accumulation of SSRBCs but not NLRBCs in the tumor blood vessels and tumor parenchyma (A,B,E,F). At the same time, SSRBC uptake is observed in the tumor vessels, there is minimal uptake in the adjacent subdermal blood vessels (C). There is also minimal uptake of NLRBCs in adjacent subdermal blood vessels (D) (Magnification 5×). Thirty minutes after infusion, the uptake of fluorescently-labeled SSRBCs (n = 5) or NLRBCs (n = 5) in tumor vessels (G) and tumor parenchyma (H) is quantitated in still video images (fluorescence intensity (FI) at Magnification 20×). SSRBCs (n = 6) show significantly greater mean FI in tumor vessels and parenchyma (G and H respectively) compared to subdermal skin vessels or NLRBCs (n = 3) (*p* = 0.00001 for FI of SSRBCs in tumor vessels and tumor parenchyma vs. respective controls in both G and H). Abbreviations in legend: *AS*: adjacent subdermal skin vessels.

**Figure 4:**
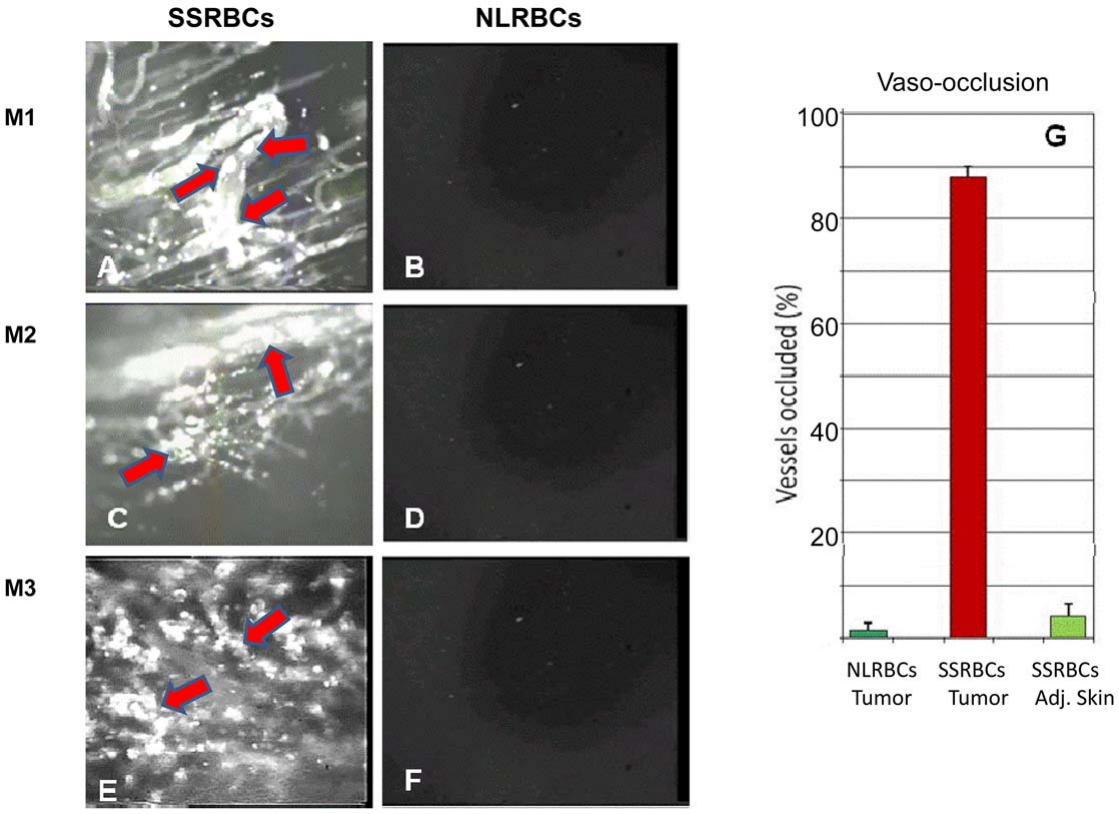
SSRBCs but not NLRBCs form microaggregates and occlude tumor microvessels. Thirty minutes after infusion of SSRBCs or NLRBCs into mice bearing eight day old 4T1 tumors, diffuse tumor vaso-occlusion is evident in mice injected with SSRBCs (A,C,E) but not NLRBCs (B,D,F). (Magnification 10× was used in panels 1–4 and 20× in panels 5 and 6). Arrows indicate SSRBC adhesion to vascular walls, microaggregate formation and partial or complete microvessel occlusion. The mice injected with SSRBCs (n = 5) showed a significantly greater percentage of occluded tumor vessels compared to NLRBCs (n = 5) or adjacent subdermal skin vessels (G) (*p* = 0.00001 for SSRBCs in tumor vessels vs. NLRBCs or adjacent subdermal skin vessels). Magnification 20× was used for quantitation of tumor microvessel occlusion. Abbreviations in legend: *Adj. skin:* adjacent subdermal skin vessels.

Using intravital microscopy with tumors implanted in dorsal skin fold window chambers, we sought to characterize the behavior of intravenously administered SSRBCs and NLRBCs in 8 day old hypoxic and neovascularized 4T1 carcinomas. Within 5 minutes after infusion, fluorescently labeled SSRBCs adhered to a large number of core and peripheral tumor microvessels (Figure 3A, Movie S1/legend). By thirty minutes, SSRBC adherence to microvessel walls increased resulting in formation of microaggregates that occluded both curved and straight segments of tumor microvessels (Figure 4A,C,E; S1/legend). Blood stasis evident at this point (Movie S1) was further substantiated by the identification of individual labeled cells on still images (Figure 4A,C,E). In the same time period, NLRBCs displayed minimal adhesion or vaso-occlusion in tumor vessels (Figure 3B; Figure 4BDF, Movie S1/legend) and neither NLRBCs nor SSRBCs showed appreciable adhesion or vaso-occlusion in adjacent normal host subdermal vascular endothelium (Figure 3C,D, Movie S1/legend).

### Quantitation of SSRBC and NLRBC tumor uptake and vaso-occlusion

To quantitate the SSRBC uptake and vaso-occlusion in tumors, compared to NLRBCs, we analyzed still images from intravital microscopic video 30 minutes after NLRBC or SSRBC infusion into mice bearing 4T1 tumors. Mice infused with fluorescently-labeled SSRBCs showed substantially greater fluorescence in tumor vessels compared to NLRBCs (p<0.005) and increased uptake in tumor vessels compared to adjacent normal subdermal vessels (p<0.008) (Figure 3G). Similarly, tumor tissues showed a 38-fold increase in RFP fluorescence of SSRBCs trapped in tumors compared to l NLRBCs (p = 0.00001) (Figure 3E,F,H). With respect to vaso-occlusion, fluorescently-labeled SSRBCs occluded 88±2.20%(SEM) of tumor vessels compared to 1.74±1.37%(SEM) for NLRBCs (p<0.00001) and 4.4±2.12%(SEM) for SSRBCs in adjacent host normal subdermal vessels (p<0.00001) (Figure 4G).

### Biodistribution of normal RBCs and SS RBCs in normal tissues and tumors

**Figure 5:**
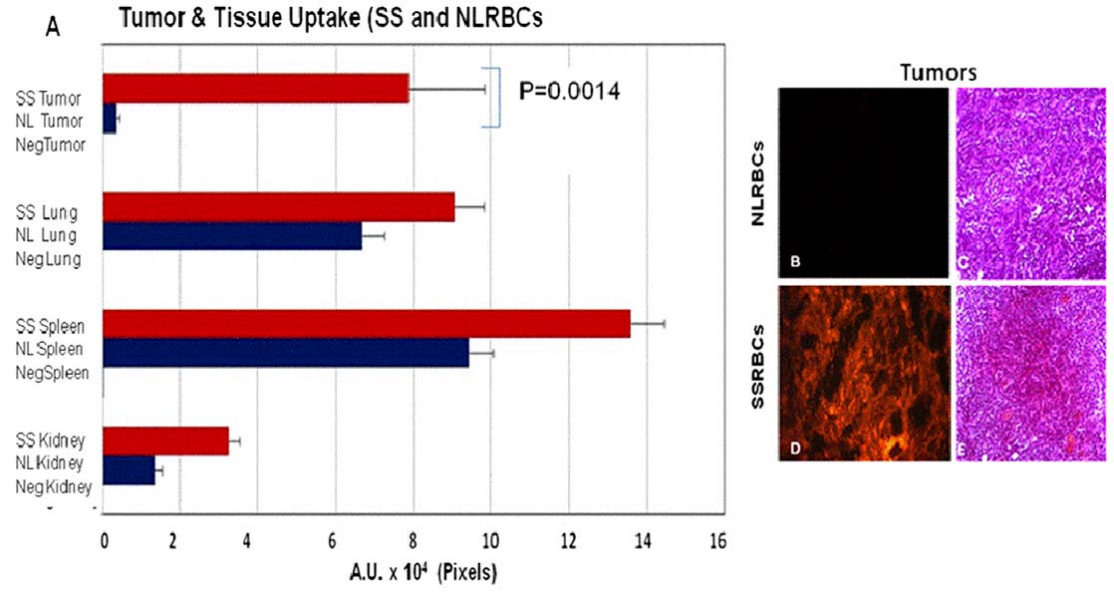
SSRBCs accumulate to a significantly greater degree in tumors compared to NLRBCs. RFP-labeled SSRBCs (n = 4) or NLRBCs (n = 2) were injected into mice bearing eight day old 4T1 tumors. Twenty four hours later tumors and organs were collected and RFP fluorescence quantitated on sections of tumors and organs. The uptake of SSRBCs in tumors is significantly greater than NLRBCs (p = 0.0014) (A, B, D). In contrast, the uptake of SSRBCs and NLRBCs is not significantly different in the spleen, lungs and kidneys (p>0.05) (A) (Magnification 5×). H&E tumor sections from SSRBC-treated mouse show focal areas of cytoplasmic eosinophilia consistent with ischemia (E) not present in tumors treated with NLRBCs (C) (Magnification 20×). Abbreviations in legend: negTumor, negLung, negSpleen, negKidney mean mice injected with NLRBCs or SSRBCs without RFP label.

We wished to determine whether the selective uptake of SSRBCs but not NLRBCs in tumors also occurred in normal organs. We therefore analyzed sections of organs and tumors harvested within 24 hours after infusion of RFP labeled SSRBCs or NLRBCs into 4T1 carcinoma for the presence of RFP-labeled SSRBCs or NLRBCs. Whereas uptake of SSRBCs in 4T1 tumors was significantly greater compared to NLRBCs (p = 0.0014), there were no significant differences in NLRBC versus SSRBC uptake in spleen, lungs or kidney (Figure 5A). Sections of tumor tissue from mice receiving SSRBCs examined at this time showed areas of cytoplasmic eosinophilia with capillary engorgement consistent with acute ischemia not seen in tumors of mice injected with NLRBCs (Figure 5E). Corresponding sections of spleen, lungs and kidneys from mice receiving SSRBCs or NLRBCs were devoid of inflammation, infarction or necrosis, indicating that SSRBCs did not induce acute injury to normal organ (Figure S2). These data indicate that SSRBC deposition in 4T1 tumor microvasculature significantly exceeds that of NLRBCs but not in other normal organs and that SSRBC uptake in tumors is accompanied histologically by acute ischemic changes but no significant pathology in normal organs.

### SSRBCs but not NLRBCs plus a pro-oxidant regimen induce a tumoricidal effect against 4T1 carcinoma in vivo

**Figure 6:**
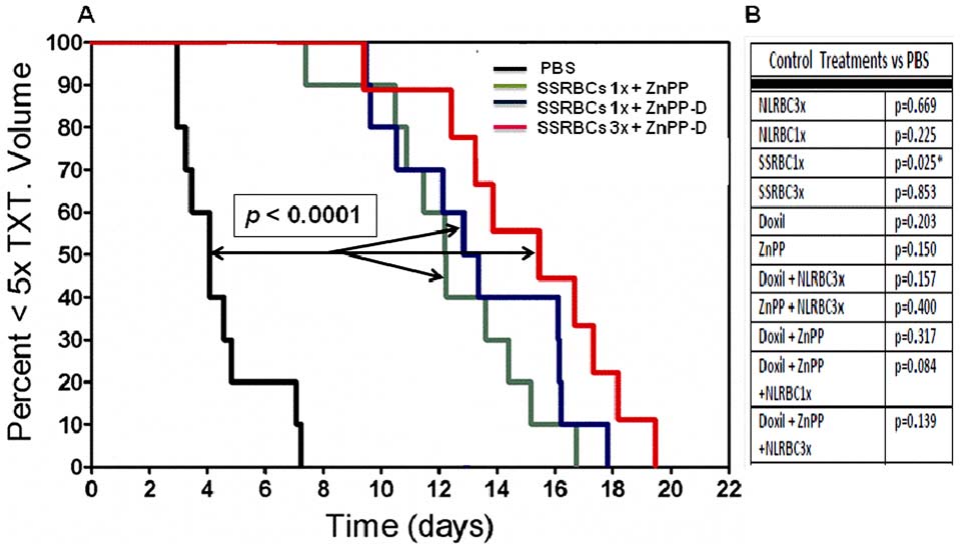
SSRBCs but not NLRBCs combined with prooxidants ZnPP and ZnPP-D induce a tumoricidal response in 4T1 bearing mice. The fraction of mice with tumors <5× the pretreatment volumes versus time is shown (n = 10 for each treatment group). All three groups treated with SSRBCs combined with ZnPP or ZnPP-D show significant tumor growth delay compared to PBS controls. In the adjacent Table, a control experiment shows that tumor bearing animals receiving SSRBCs 1× or 3× alone, NLRBC 1× or 3× alone, NLRBCs 1× or 3× with ZnPP or ZnPP-D, ZnPP alone or Doxil + ZnPP exhibited no significant tumor growth delay versus the PBS control. * indicates that mice receiving SSRBC1× alone displayed significantly accelerated tumor growth compared to the PBS control.

Studies in sickle cell patients have shown that SSRBCs involved in postcapillary occlusion can release SSRBC-derived heme, hemichrome and ROS causing oxidative tissue damage [35]–[41]. We therefore determined whether SSRBCs could induce a tumoricidal response when the tumor cells are rendered susceptible to oxidative stress with exogenous pro-oxidants. For this purpose, we deployed two potent pro-oxidative regimens, zinc protoporphyrin (ZnPP) (a competitive inhibitor of heme oxygenase) alone or together with doxorubicin (ZnPP-D). Both regimens were shown previously to efficiently promote cytotoxicity of tumor cells exposed to oxidative stress in vitro in an NADH-dependent manner [42],[43]. Groups of 10 mice with established 4T1 tumors were treated with one or three SSRBC infusions plus ZnPP or ZnPP-D (see Table S1 for protocol). This resulted in a dramatic delay in tumor growth compared to the PBS control group (*p*<0.0001) (Figure 6A). Notably, the group receiving three SSRBC infusions showed a quadrupling of growth delay compared to PBS control. In contrast, tumor bearing mice receiving SSRBCs1x or 3x or NLRBCs1x or 3x, ZnPP alone, Doxil alone or combinations of NLRBCs3x with ZnPP, NLRBCs1x or 3x with Doxil or ZnPP-D induced no growth delay compared to the PBS control (Figure 6B). Since SSRBCs1x or 3x show potent anti-tumor effects when used in combination but are ineffective by themselves, SSRBCs together with ZnPP or ZnPP-D appear to exhibit a mutual potentiation. Mice displayed no acute toxicity of SSRBC1x or 3x combined with ZnPP or ZnPP-D and there were no significant differences in weights compared to the PBS control group (*p* = 0.485). H&E sections from mice treated with SSRBC + ZnPP-D showed more diffuse tumor necrosis than PBS controls. Spleens of mice treated with SSRBCs3x + ZnPP-D displayed scattered hemosiderin deposits not present in untreated control tissues. However, liver, kidney, spleen and brain, including the hippocampus, cortex, cerebellum and Purkinje fibers, from SSRBC-treated and PBS controls were unremarkable and notably devoid of SSRBC vascular aggregates, inflammation, infarction and necrosis. Thus, treatment with SSRBC3x + ZnPP-D did not induce histologically demonstrable toxicity in normal host organs.

### Sickle cell oxidants induce 4T1 cell death in the presence of pro-oxidants in vitro

**Figure 7:**
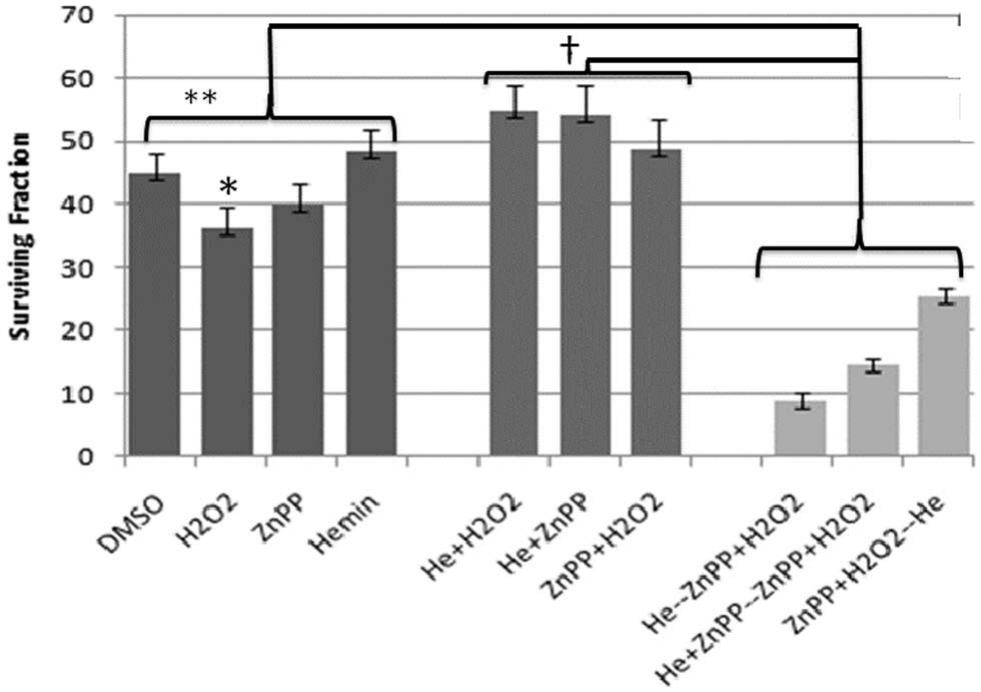
Tumoricidal effect of the combination of hemin, H_2_O_2_ and ZnPP in a clonogenic tumor survival model. Three agent regimens consisting of pre-treating 4T1 cells with i) hemin alone or combined with ZnPP for 2 hrs followed by the combination of ZnPP and H_2_O_2_ for 2 hrs or, ii) ZnPP for 2 hours followed by the combination of hemin and H_2_O_2_ for 2 hours induced significant tumor cell death compared to each agent individually (**p<0.0002) and any two of these agents used simultaneously (†*p*<0.0001). Clonogenic survival is shown as a mean of three independent experiments with standard error (SE) indicated. See Table S2 for protocol used in these studies.

We sought to understand the mechanism of the mutual potentiation between SSRBCs and ZnPP in the tumoricidal effect noted *in vivo*. In the course of vascular adhesion, entrapped SSRBCs generate pro-oxidant membranes along with oxidized hemichrome and activated endothelial cells produce hydrogen peroxide (H_2_O_2_) [36],[38],[41]. We reasoned that tumor cells deprived of oxidant protection by heme oxygenase inhibition would be susceptible to apoptosis. To test this hypothesis, we used a clonogenic tumor cell survival model in vitro in which we exposed 4T1 cells to heme oxygenase inhibition (ZnPP) [44], hemin [protoporphyrin IX containing ferric iron (heme b)] and H_2_O_2_ alone and in various combinations (see Figure S2 for protocol). Incubation of 4T1 tumor cells with hemin, H_2_O_2_ or ZnPP alone resulted in no significant tumor killing (p>0.5). Likewise, using hemin together with H_2_O_2_ or ZnPP did not enhance the tumor cell killing (p>0.05) (Figure 7). In contrast, treating 4T1 cells with i) hemin alone or combined with ZnPP for 2 hrs followed by ZnPP and H_2_O_2_ for 2 hrs or, ii) ZnPP for 2 hours followed by hemin and H_2_O_2_ for 2 hrs resulted in significantly increased 4T1 tumor cell killing compared to each agent alone (p<0.0002) or any two of these agents used simultaneously (p<0.0001) (Figure 7). Since only the three agent regimen (ZnPP, hemin and H_2_O_2_) used simultaneously or sequentially produced a significant tumoricidal effect whereas the individual agents were ineffective, these data are concordant with the mutual potentiation of SSRBCs and ZnPP in the tumoricidal response described above *in vivo* in the 4T1 tumor model.

## Discussion

Resistance of hypoxic solid tumor niches to chemotherapy and radiotherapy remains a major scientific challenge that invites conceptually new approaches. Here we exploit the previously unrecognized ability of the sickled erythrocyte (SSRBC), but not NLRBCs, to selectively target hypoxic microvessels of solid tumors and induce diffuse tumor vascular occlusion. Importantly, SSRBCs, but not normal RBCs, also induce a potent tumoricidal response via a mutual potentiation of exogenous pro-oxidants ZnPP or ZnPP-D. A clonogenic tumor cell survival model confirms this mutual potentiation and demonstrates a key obligate role for SSRBC-derived heme and H_2_O_2_ in potentiating the tumoricidal effect of ZnPP. In addition to SSRBC's remarkable tumor targeting ability, this is the first report that harnesses the SSRBC for anti-tumor therapy.

In contrast to pharmaceutical treatment directed only to the hypoxic tumor cell, the present approach targets the hypoxic tumor vascular microenviroment and induces injury to hypoxic tumor microvessels and tumor cells using intrinsic SSRBC oxidants and locally generated ROS. Data in Figures 3 and 4 and their legends plus the Movie S1 demonstrate that the initial events in the tumoricidal process consist of rapid adherence of SSRBCs but not NLRBC to tumor vasculature, formation of microraggregates leading to diffuse tumor microvessel occlusion. The SSRBC adherence to tumor microvessels could not be ascribed to non-specific RBC trapping since NLRBCs rarely adhered to tumor vessels. Likewise, it could not be attributed to asymmetric distribution of SSRBCs cells and NLRBCs in the host, since NLRBCs and SSRBCs sequester in normal organs to a similar degree.

Concurrent with SSRBC localization, adherence and occlusion of tumor microvessels, our data further demonstrate the presence hypoxia in the 4T1 tumor microenvironment. Hyperspectral imaging indicates that 70% or the 4T1 tumor microvessels exhibit a hemoglobin saturation between 0–10% which corresponds to pO_2_ values <10% mm Hg in the oxygen-hemoglobin dissociation curve [45],[46]. This degree of tumor hypoxia [8],[47] could prime the tumor vasculature for adherence/vaso-occlusion by SSRBCs by stimulating tumor cell synthesis of proangiogenic/pro-inflammatory proteins such as VEGF and TNFα [1],[2],[24],[26]. The latter upregulates several tumor vascular adhesion molecules capable of capturing activated SSRBCs [23],[24],[27]–[30]. Figure 2 demonstrates that several such vascular adhesion ligands are expressed in tumor microvessels. As in painful sickle cell crisis, the inflammatory/procoagulant condtions at the site of SSRBC-inuduced tumor vaso-occlusion could also promote platelet activation and leukocyte recruitment leading to accelerated vascular injury [26].

**Figure 8:**
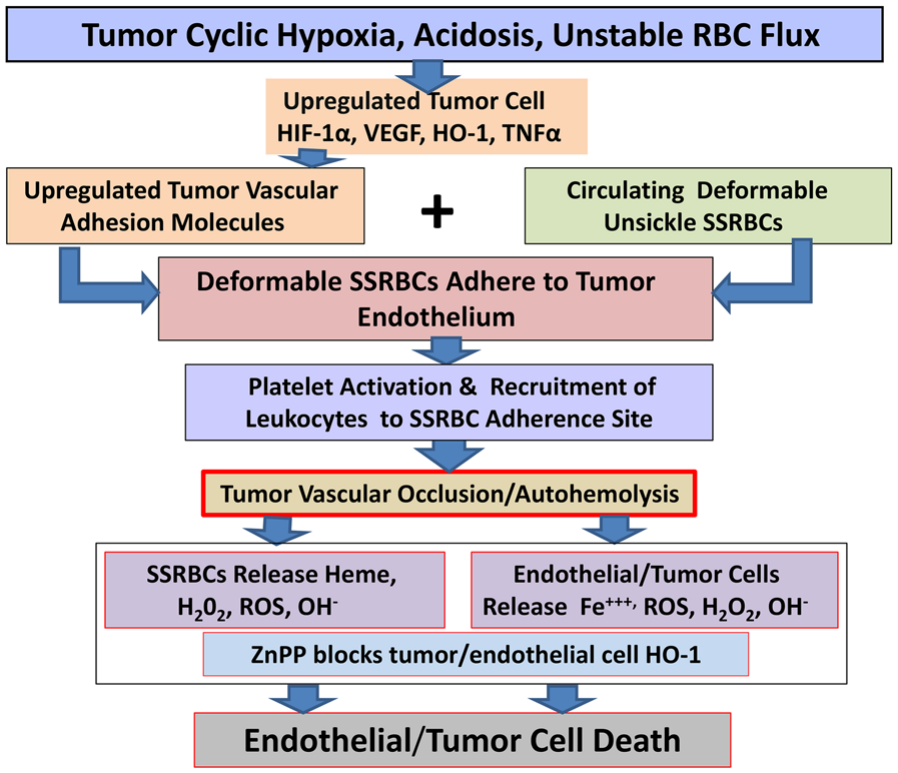
Schematic depiction of proposed pathophysiology of tumor killing induced by SSRBCs and the HO-1 inhibitor ZnPP. The hypoxic and acidic tumor milieu activates HIF1α, which, in turn, stimulates VEGF and HO-1 expression and the production of TNFα. TNFα upregulates several adhesion molecules on tumor endothelium, including several endothelial cognate adhesion ligands for the major adhesion receptors expressed on SSRBCs. Deformable non-sickled SS RBCs adhere to the activated endothelium of the tumor vasculature, along with leukocytes to form microaggregates leading to tumor vascular obstruction/occlusion. Entrapped SSRBCs release SS hemoglobin which is converted rapidly to methemoglobin and cleaved to liberate free heme. Hydrophobic and lipophilic heme and/or heme-nitrosyl complexes permeate tumor and endothelial cell membranes where they catalytically oxidize lipids, proteins and DNA causing cell death. In the presence of ZnPP, a competitive inhibitor of HO-1, intracellular heme and oxidative products such as reactive oxygen and nitrogen species (ROS and RNS) are free to exert their potent oxidative function leading to tumor and endothelial cell death.

Our proposed mechanism of the tumoricidal effect in this system, shown schematically in Figure 8, implicates SSRBCs-induced tumor vaso-occlusion in hypoxic tumor vessels as the central event in both tumor vascular endothelial cell and tumor cell injury. We hypothesize that SSRBCs entrapped in the vaso-occlusive process undergo autohemolysis and release intrinsic hemichrome, hemoglobin S and ROS. These powerful cellular toxins are capable of inducing tumor endothelial cell and tumor cell injury [35]–[40]. SSRBC hemichrome, for instance, spontaneously generates twice as much superoxide, peroxide/hydroxyl radicals as NLRBCs [35],[38] and hemoglobin S is rapidly converted to methemoglobin which forms highly lipophilic heme-nitrosyl complexes that intercalate and oxidize cell membranes [48]. Tumor endothelial cells activated by SSRBCs contribute to the process by generating hydrogen peroxide, leading to endothelial membrane injury (peroxidation) and diapedesis of inflammatory monocytes into the tumor parenchyma [19],[41].

We further surmised that the effectiveness of these SSRBC/endothelial cell-derived oxidants is enhanced when the tumor and endothelial cells are exposed to ZnPP, a metalloporphyrin that competitively inhibits the degradation of heme by heme oxygenase [49]. The latter, a 32-kD microsomal membrane enzyme is overexpressed in the 4T1 tumor and a broad array of tumor cell types [49]. Indeed, these studies show that SSRBC infusion combined with ZnPP exhibited a unique mutual potentiation in tumor killing *in vivo*. This novel effect was mimicked in our clonogenic tumor survival model *in vitro* wherein hemin and H_2_O_2_., surrogates for SSRBC-derived hemichrome and endothelial cell H_2_O_2_ respectively, efficiently potentiated the death of ZnPP-treated 4T1 tumor cells.

The accelerated tumor growth noted after a single SSRBC infusion was reversible after the addition of ZnPP, a potent inhibitor of HO-1. This effect may be ascribed to SSRBC-induced vaso-occlusion in the tumor resulting in increased tumor hypoxia and release of SSRBC-derived heme. Both heme and hypoxia activate HIF-1α in tumor cells which, in turn, stimulates synthesis of HO-1 [50]. The latter is a powerful stimulant of tumor angiogenesis and promotes tumor growth [49]. Since the addition of ZnPP to SSRBCs abrogated the accelerated tumor growth and promoted statistically significant growth delay, HO-1 may be pivotal in the accelerated tumor growth process induced by SSRBCs alone. In clinical translation, it would appear that SSRBCs should be administered with caution and preferably together with prooxidant agents to avoid promoting tumor growth.

Importantly, mice infused with SS RBCs and ZnPP or ZnPP-D showed no significant organ toxicity, and body weights were stable throughout the study. Only the tumor showed extensive necrosis, whereas spleen, liver, brain, lungs and kidneys from the treated mice were devoid of infarction, inflammation and necrosis. Thus, it appears that the cytotoxic activity of SSRBC/ZnPP-D is selective for the tumor but not normal tissues. As a source of SSRBCs for clinical use, nucleated sickle progenitor cells (phenotypic and functional sickle cells) can be readily expanded/differentiated in vitro. Moreover, nucleated SS progenitors can also be transduced with virtually any tumoricidal transgene. Thus, the SSRBC appears to be a potent and versatile new tool for treatment of hypoxic solid tumors notable for their resistance to existing cancer treatments.

## Methods

### Mice

All animal experiments were approved by the Duke University ACUC. Female athymic homozygous nude mice (nu-/nu-), between 8–12 weeks of age weighing 19–26 grams, obtained from Charles River Laboratories (Wilmington, MA) or Harlan Laboratories (Indianapolis, IN) were used for all experiments. The animals were housed 5 animals per cage in a 12 h light-dark cycle with water, food *ad libitum*. All infusions were performed using the tail vein.

### Cell lines, virus and reagents

The 4T1 murine mammary carcinoma, a thioguanine and doxorubicin resistant tumor [51], [52], were cultured with DMEM supplemented with 10% (v/v) fetal bovine serum (and 1% (v/v) antibiotics-antimycotics as previously described [53]. All cell lines were monitored routinely and found to be free of mycoplasma infection. Fresh solutions of hemin (Sigma) in DMSO and Zn (II) Protoporphyrin IX (Frontier Scientific) in 50% DMSO-50% 0.1 M NaOH was made for each experiment. H_2_O_2_ was purchased from (Sigma-Aldrich, St. Louis, MO).

### Western blotting analysis of HO-1 expression

For immunoblotting, proteins were extracted from snap frozen mouse tumor, kidney, and liver tissues. Tissues were homogenized and dissolved in the cold RIPA buffer (Pierce, Rockford, IL). Cell debris was separated by centrifuging twice at 10,000 g for 10 min at 4°C. Whole protein concentration was measured by Bradford assay (Bio-Rad Laboratories, Hercules, CA). Each Protein preparation (100 µg) was electrophoresed in 12% sodium dodecyl sulfate-polyacrylamide gel electrophoresis (SDS-PAGE). Proteins were transferred to PVDF (polyvinylidene fluoride) membrane and blocked for 1 hour with 5% non-fat dried milk in TBST (20 mM Tris-HCl, 150 mM NaCl, and 0.1% Tween 20, pH 7.5). Membranes were incubated overnight with 1∶500 diluted anti-mouse HO-1 antibodies (Assay Designs, Ann Arbor, Michigan), washed three times with TBST and incubated with 1∶1000 diluted horseradish peroxidase (HRP)-conjugated anti-mouse IgG antibody for 1 hour at room temperature followed by washing with TBST. The blot was immunodetected with enhanced chemiluminescence (ECL) detection system (Perkin Elmer, Waltham, Massachusetts). For a loading control, 50 µg of protein was loaded in 10% SDS-PAGE and blotted with 1∶2000 diluted anti-mouse β-actin antibody (Sigma-Aldrich).

### Immunohistochemical localization of adhesion molecules in tumor microvessels

Frozen tumor tissues were sectioned at 10 micron thickness and were kept at −80°C until the immunohistochemistry was performed (n = 5). Before staining, frozen tissue sections were air-dried for 30 minutes and fixed for 10 minutes in cold acetone. Tissues were air-dried again for 30 minutes and incubated with phosphate-buffered saline (PBS) for 5 minutes. After 30-minute blocking with 10% serum, tissues were incubated overnight at 4°C with primary antibodies, CD31 (PECAM-1), CD106 (VCAM-1), CD51 (Integrin av) all from BD Pharmingen and laminin α5, a kind gift from Dr. Jeffrey H. Miner, Washington University, St. Louis. Slides were then washed three times in PBS for 5 min followed by the incubation with the appropriate secondary antibody (Jackson Immuno-Research, West Grove, PA) for 30 min at room temperature. Again slides were washed three times in PBS for 5 min followed by incubation with ABC-Elite (Vector Laboratories, Burlingame, CA) for 30 min at room temperature. Reaction was localized by using 3.3′-diaminobenzidine tetrachloride (DAB) working solution (Laboratory Vision, Fremont, CA). Finally, the slides were counterstained with Harris haematoxylin (Fisher Scientific, Pittsburgh, PA) and mounted with coverslips. For image analysis the slides were systematically scanned with a light microscope (Zeiss Axioskop 2 plus, Oberkochen, Germany) and digital images were acquired from each slide using 5×, 10×, and 40× objectives using the software (Axiovision 3.1).

### Collection, preparation and treatment of human RBCs

NLRBCs were obtained from normal healthy adults or SSRBCs from homozygous SS patients with approval by the Institutional Review Board and Ethics Committees of Duke University and informed consent was obtained from each donor. All participants provided their written informed consent to participate in this study which was documented by two witnesses. Fresh blood samples from patients homozygous for hemoglobin S and from normal controls were collected into citrate tubes. RBCs were allowed to separate from the buffy coat containing leukocytes and platelet-rich plasma by gravity at 4°C for at least 2 h. Plasma and buffy coat were removed by aspiration and RBCs were washed four times in sterile PBS with 1.26 mM Ca^2+^, 0.9 mM Mg^2+^ (pH 7.4). Packed RBCs were analyzed for leukocyte and platelet contamination using an Automated Hematology Analyzer Sysmex K-1000 (Sysmex, Co., Cobe, Japan). Packed RBCs were fluorescently labeled with DiI (Molecular Probes, Eugene, OR) for *in vivo* uptake studies as previously described [54],[55]. Dil has no effect on RBC suspension viscosity or RBC survival in the circulation [54]. Cells were then washed three times with 5 ml PBS with Ca^2+^ and Mg^2+^.

### Window chamber surgery and murine mammary carcinoma implantation

This procedure has been described previously [56]. General anesthesia was induced by intraperitoneal (IP) injection of 100 mg/kg of ketamine (Abbott Laboratory, Chicago, IL) and 10 mg/kg of xylazine (Bayer, Shawnee Mission, KS). A double-sided titanium frame window chamber was surgically implanted into the dorsal skin fold under sterile conditions with aseptic technique. Surgery involved carefully removing the epidermal and dermal layers of one side of a dorsal skin fold, exposing the blood vessels of the subcutaneous tissue adjacent to the striated muscles of the opposing skin fold. The two sides of the chamber were secured to the skin using stainless steel screws and sutures, followed by injection of 1×10^4^ 4T1 tumor cells into the exposed fascia. A glass window was placed in the chamber to cover the exposed tissue, and was secured to the chamber with a snap ring. Animals were kept in a specialized environmental chamber at 32–34°C and 50% humidity until in vivo studies were performed 8 days post-surgery.

### Intravital microscopy and visualization of RBC trafficking

The set up for window chamber visualization was identical to that described above. Labeled human NLRBCs or SSRBCs (300 µL; hematocrit 50% in PBS with Ca^2+^ and Mg^2+^) were infused through the tail vein and blood flow dynamics were observed in both tumor neovasculature and subdermal vessels for at least 30 minutes, using LD Achroplan 20X/0.40 Korr and Fluar 5X/0.25 objectives (Zeiss). Microcirculatory events and cell adhesion were simultaneously recorded using a Trinitron Color video monitor (model PVM-1353 MD, Sony) and JVC videocassette recorder (model BR-S3784, VCR King, Durham, NC) connected to a digital video camera C2400 (Hamamatsu Photonics K.K., Japan). Blood vessels were also viewed under fluorescence-illumination using a 100-W mercury arc lamp and 5× and 20× magnifications.

Hemoglobin saturation determinations in the 4T1 tumor microvasculature using hyperspectral imaging information was described previously [57]. A Zeiss Axioskop 2 microscope (Carl Zeiss, Inc., Thornwood, NY) served as the imaging platform. Images were acquired with a CCD camera (DVC Company, Austin, TX), and bandlimited optical filtering for hyperspectral imaging was accomplished with a C-mounted liquid crystal tunable filter (CRI, Inc., Woburn, MA). Image processing was performed using Matlab software (The Mathworks, Inc., Natick, MA). Microvessel-based pixel counts of vessels in window chamber tumors were quantitated as a fraction of microvessels pixels with hemoglobin saturations of 10% or less over the total number of micropixels in the tumor as described [57].

Quantification of vaso-occlusion was performed by examining videotapes using 20× magnification. Multiple segments of tumor and adjacent normal subdermal microvessels were examined 30 minutes following SS RBC and normal RBC infusions. Vessels were counted as occluded by considering labeled cells attached to the vessel walls and immobile for at least 10 seconds with no observable blood flow. The percentage of vessels occluded by SS or normal RBCs was calculated by division of the number of occluded vessels by the total number of vessels in the field that contained visible blood flow at baseline.

### Histology

The animals used in window chamber experiments were sacrificed 30 minutes post-injection of Dil-labeled RBCs. Tumors and organs were collected and snap frozen in OCT media. Forty micron sections were cut from four standardized locations in each organ mounted and examined via inverted fluorescence microscopy. Three random fields were imaged for each section of each organ. RBC fluorescence intensity for each field was quantified using Adobe Photoshop CS2 software (Adobe Systems Inc., San Jose, CA). Five determinations of pixel intensity were obtained for each field and averaged for the three fields to obtain mean fluorescence intensity. The mean fluorescence values were averaged among groups of animals for statistical analysis. In tumor therapy studies, tumors, organs and brain from hippocampus, cortex, cerebellum and Purkinje fibers were collected in 4% paraformaldehyde or 10% formalin and stained using hematoxylin and eosin and Prussian blue.

### Tumor therapy studies

All procedures were approved by the Duke University Institutional Animal Care and Use Committees or the Animal Use Committees in compliance with the Guide for the Care and Use of Laboratory Animals. For the studies in mice bearing 4T1 carcinoma, tumor volume and body weight were measured every 2 days, and volumes were calculated as π/6*length^2^*width. The treatment endpoint was 5× treatment volume or 1500 mm^3^, whichever was reached first. Zinc (II) protoporphyrin IX (Zn-PP; Frontier Scientific) was dissolved in a solution of saline and N,N dimethylformamide (DMF) at a 95/5 volume ratio to a concentration of 0.1 mg/ml prior to i.p. injection. Lyophilized Doxorubicin (DOX; Bedford Laboratories) was hydrated with saline (2 mg/ml) prior to i.v. administration. Study groups consisted of 10 mice per cohort. Treatments were started when the tumors were at a median volume of 72 mm^3^ (57–90 mm^3^ interquartile range). SSRBCs were infused iv in 150–200 µl, hematocrit 50%, Zn-PP, 0.5 mg/kg, was injected i.p. and Doxorubicin, 5 mg/kg, was administered. i.v. on a schedule shown in Table S1. Tumors were measured twice a week with standard calipers and mice were monitored for toxicity. Mice were euthanized if toxicity was evident or tumor burden exceeded 1500 mm^3^.

### Biodistribution studies

RFP-labeled SSRBCs or NLRBCs (300 µL; hematocrit 50% in PBS with Ca^2+^ and Mg^2+^) were infused through the tail vein into atyhmic nude mice bearing eight day old 4T1 tumors. Twenty four hours later tumors and organs were collected. RFP fluorescence was quantitated as described below at magnification 5× using Adobe Photoshop CS2 software (Adobe Systems Inc., San Jose, CA). Five to ten determinations of pixel intensity were obtained for each field and averaged for the three fields to obtain mean fluorescence intensity. The mean fluorescence values for tumors and organs in each group were used for statistical analysis.

### Clonogenic survival assays

4T1 were plated in 6-well plates at two different densities (100 and 300 cells per well) in 3 ml of media and allowed to attach for 24 hr at 37°C. For the individual treatments, cells were treated for 2 hr with DMSO (vehicle), hemin (100 µM), H_2_O_2_ (100 µM), or the Zn (II) protoporphyrin IX (ZnPP) (10 µM). For the combination treatments, cells were treated with the indicated agents for 2 hr, which were then removed and followed by treatment with the other agents for an additional 2 hr. After the last treatment, the media containing drugs was removed and the cells incubated in fresh media at 5% CO_2_ and 37°C for 7–10 days. After incubation, cells were fixed with 10% methanol-10% acetic acid and stained with a 0.4% crystal violet solution. Colonies with >50 cells were counted using a ColCounter (Oxford Optronix). Plating efficiencies were determined for each treatment and normalized to controls. The data shown in Figure 7 is the mean of 3 independent experiments. The protocol for this study is shown in Table S2.

### Statistics

All data analysis was performed using GraphPad Prism version 4.03 for Windows (GraphPad Software, San Diego California USA). Kaplan-Meier analysis was used to evaluate treatment efficacy for 4T1 carcinoma studies. Statistical comparison were performed with Kruskal-Wallis or log rank test. For multiple comparisons in the 4T1 study, p<0.01 was considered the threshold for significance. In clonogenic tumor survival studies, the plating efficiency was 0.4 for 4T1 cells and 4T1 cell survival was normalized to the DMSO control. Two way ANOVA was used to compare surviving fraction of cells among independent treatment groups with treatment and experiment as categorical independent factors. Specific contrasts by F test under linear models were used to assess the treatment difference between groups with type I error adjusted to 0.01. Window chamber/histology data, starting tumor volume size, maximum weight change were analyzed using either student T-test or Kruskal-Wallis for multiple group comparisons.

## Supporting Information

Figure S1 **Expression of heme oxygenase-1 in tumor and normal tissues.** Western blots of protein extracted from 4T1 tumor, normal liver and kidney, stained for heme oxygenase-1 (HO-1). Increased expression of HO-1 in the tumor was observed compared to the kidney and liver tissues. For a loading control, proteins were blotted with an anti-mouse β-actin antibody.
(TIF)

Figure S2 **H&E sections of organs from 4T1 bearing mice 24 hours post RBC infusion.** H&E sections of lung, spleen and kidney from mice infused with SSRBCs (n = 5) or NLRBCs (n = 3) 24 hours post SSRBC or NLRBC infusion were unremarkable and notably devoid of inflammation, infarction or necrosis. (Magnification10×)
(TIF)

Movie S1 **Intravital microscopy of the skin window of mice using 8 day old 4T1 carcinoma infused with SSRBCs or NLRBCs.** SSRBC-1: Five minutes after infusion of SSRBCs, the SSRBCs are adherent to vascular walls and deposited in relatively avascular core (circular, dark area at top) and periphery of the tumor microvasculature. Microcapillary obstruction is evident along with reduced velocity of SSRBCs as they transit through partially obstructed vessels. At 20 minutes, SSRBC microaggregates have formed in the vessels several of which have progressed to frank vaso-occlusion (Magnifications 10×). At 30 minutes, extensive aggregate formation and vaso-occlusion is noted along with retrograde blood flow in a patent capillary segment within surrounding occluded microvessels (Magnifications 20×). **NL**RBCs depicted 30 minutes after infusion into mice with 8 day old 4T1 carcinoma exhibit minimal adherence to tumor microvessels with no evidence of vaso-occlusion (Magnifications 10×).
(MOV)

Table S1 Schedule of treatments.
(TIF)

Table S2 Clonogenic tumor model protocol.
(TIF)
