# Supplementary figures and images for: Sickle Erythrocytes Target Cytotoxics to Hypoxic Tumor Microvessels and Potentiate a Tumoricidal Response
Source: PLoS One. 2013 Jan 9;8(1):e52543. doi: 10.1371/journal.pone.0052543 (PMC3541382; doi:10.1371/journal.pone.0052543)

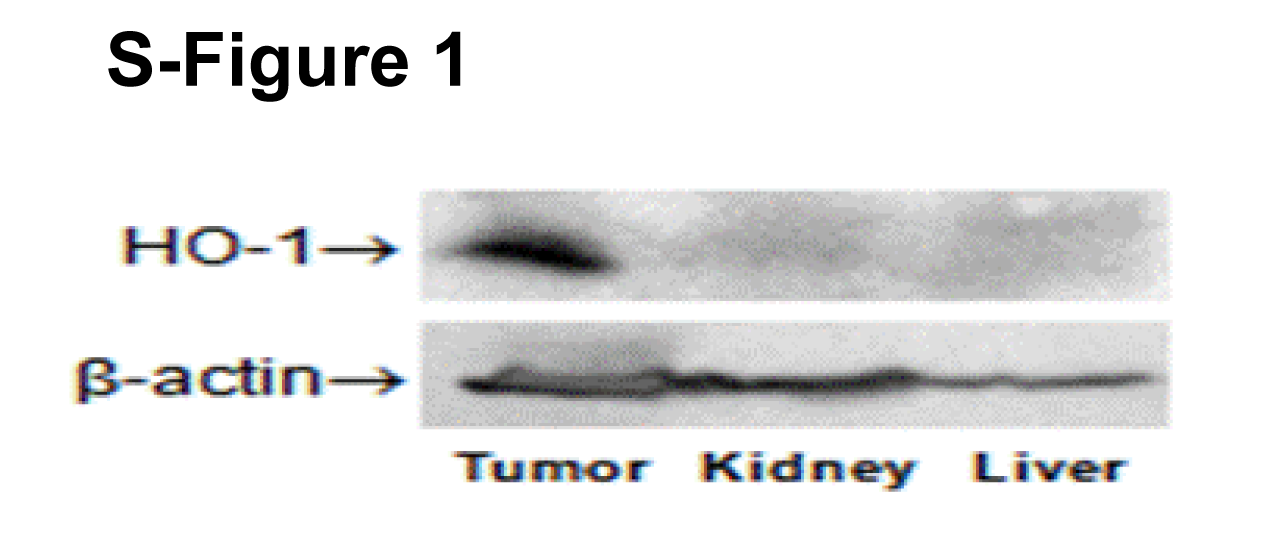

Supplement: Figure S1 — Expression of heme oxygenase-1 in tumor and normal tissues. Western blots of protein extracted from 4T1 tumor, normal liver and kidney, stained for heme oxygenase-1 (HO-1). Increased expression of HO-1 in the tumor was observed compared to the kidney and liver tissues. For a loading control, proteins were blotted with an anti-mouse β-actin antibody. (TIF) [file pone.0052543.s001.tif]

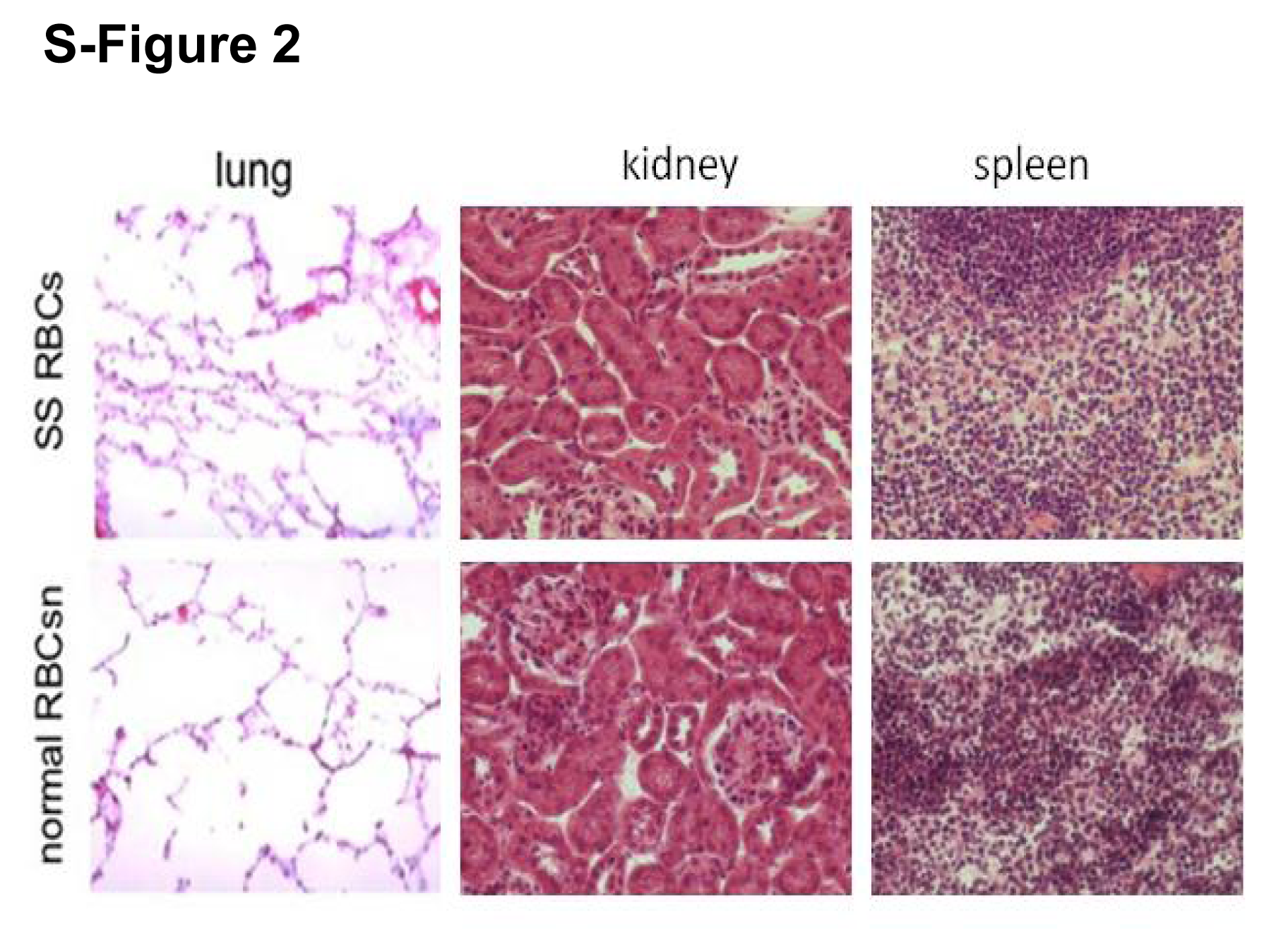

Supplement: Figure S2 — H&E sections of organs from 4T1 bearing mice 24 hours post RBC infusion. H&E sections of lung, spleen and kidney from mice infused with SSRBCs (n = 5) or NLRBCs (n = 3) 24 hours post SSRBC or NLRBC infusion were unremarkable and notably devoid of inflammation, infarction or necrosis. (Magnification10×) (TIF) [file pone.0052543.s002.tif]

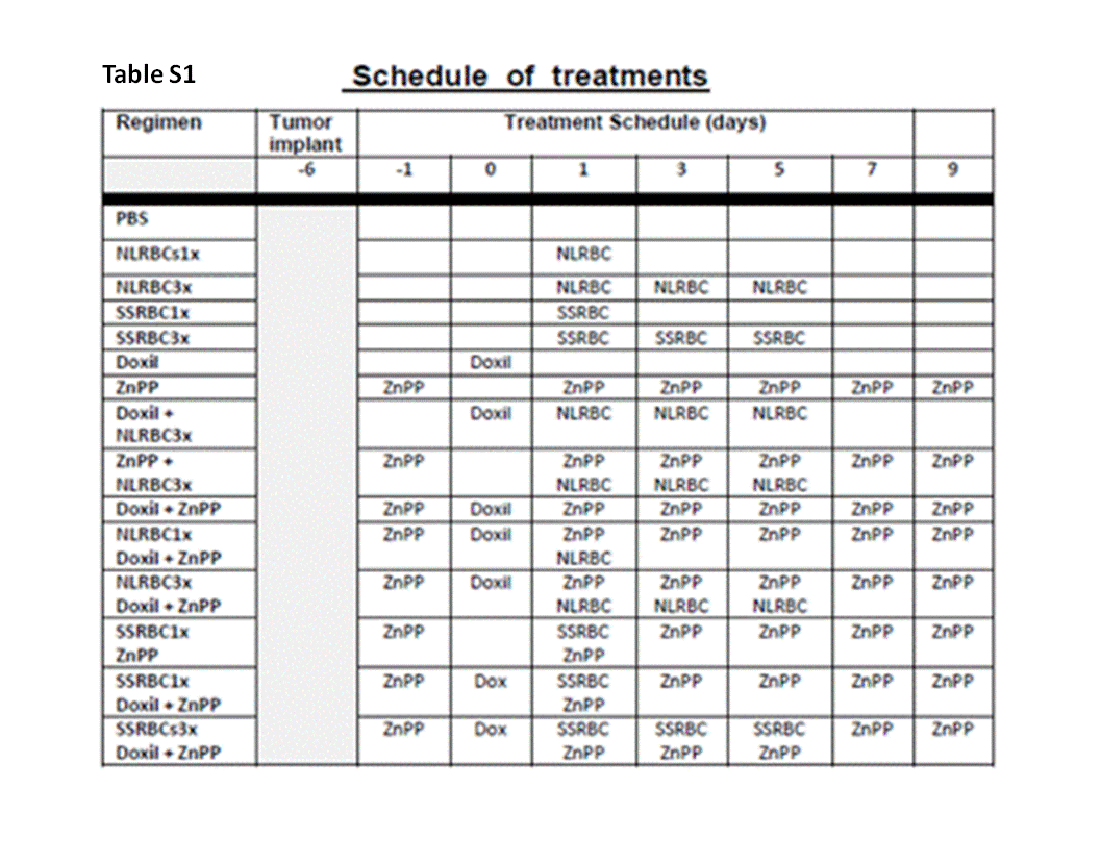

Supplement: Table S1 — Schedule of treatments. (TIF) [file pone.0052543.s004.tif]

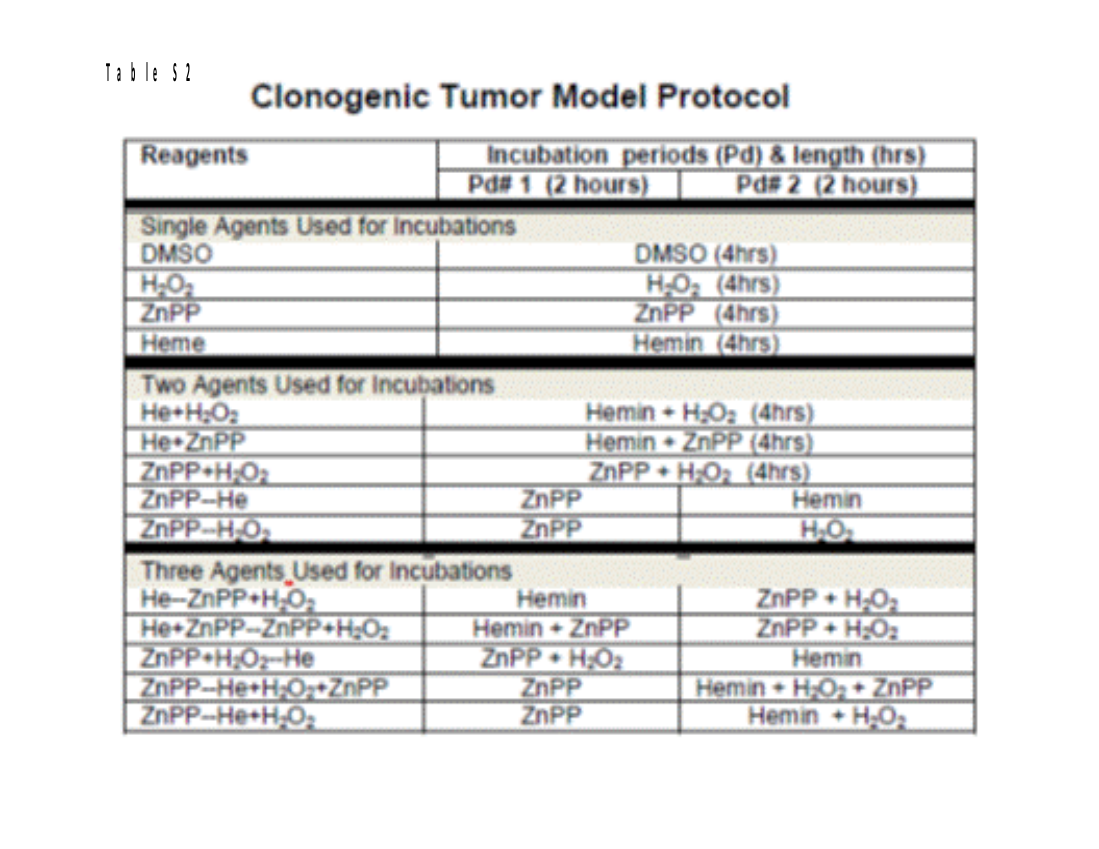

Supplement: Table S2 — Clonogenic tumor model protocol. (TIF) [file pone.0052543.s005.tif]
